# Reversible Fetal Renal Impairment following Angiotensin Receptor Blocking Treatment during Third Trimester of Pregnancy: Case Report and Review of the Literature

**DOI:** 10.1155/2016/2382031

**Published:** 2016-09-08

**Authors:** Tal Saar, Lorinne Levitt, Hagai Amsalem

**Affiliations:** Hadassah University Hospital Mount Scopus, Jerusalem, Israel

## Abstract

*Background*. Late pregnancy usage of angiotensin converting enzyme inhibitors (ACE-I) and angiotensin II receptor blockers (ARB) may cause severe oligohydramnios due to fetal renal impairment. Affected neonates will often suffer from fatal, renal, and respiratory failure.* Case*. A 39-year-old multigravida admitted due to anhydramnios secondary to valsartan (ARB) exposure at 30 weeks' gestation. Following secession of treatment amniotic fluid volume returned to normal. Delivery was induced at 34 weeks' gestation following premature rupture of membranes and maternal fever. During the two-year follow-up, no signs of renal insufficiency were noted.* Conclusions*. This description of reversible fetal renal damage due to ARB intake during pregnancy is the first to show no adverse renal function in a two-year follow-up period. This case may help clinicians counsel patients with pregnancies complicated by exposure to these drugs.

## 1. Introduction

Chronic hypertension is a relatively common condition, affecting an estimated 7% of reproductive age women [1]. More than half of these women will conceive while taking antihypertensive medication. Common pharmacological treatment includes angiotensin converting enzyme inhibitors (ACE-I) and angiotensin II receptor blockers (ARB). These medications have been linked to adverse perinatal outcomes since Cooper's first epidemiologic report of first trimester exposure to ACE-I [2].

Cooper et al. [2] described a relative risk of 2.7 for major congenital anomalies, primarily cardiovascular, with first trimester ACE-I exposure. Several other population based studies have linked all classes of antihypertensive medications to congenital anomalies [3, 4]. Recently, a large population based cohort study suggested that hypertension itself, rather than its treatment, is responsible for this increased risk of congenital anomalies [5].

While it remains inconclusive whether first trimester exposure to ACE-I or ARB poses a risk for congenital anomalies, it is quite apparent that second and third trimester exposure is related to adverse perinatal events, mostly due to the effect on renal function [6]. In utero, these drugs may cause severe oligo- or even anhydramnios resulting in fetal heart rate abnormalities during labor or intrauterine fetal demise, secondary to cord compression. Severe oligohydramnios occurring prior to 22 weeks' gestation may also lead to pulmonic hypoplasia and limb contractures due to abnormal fetal posture. Postnatal affected newborns may suffer from oliguria, renal, and respiratory failure [7, 8]. It is not known as to what extent this fetal renal impairment, occurring during the third trimester, can be reversed following cessation of treatment.

We describe a case of anhydramnios secondary to ARB (valsartan) treatment, diagnosed during the third trimester. Following cessation of treatment, amniotic fluid volume returned to normal. Neonatal and infant renal function remained normal throughout the two-year follow-up period. A “PubMed” search yielded only few case reports describing reversible fetal renal insufficiency [9–11] with only short term follow-up.

## 2. Case

A 39-year-old woman, gravida 13 para 3, was admitted to the High Risk Pregnancy Unit at Hadassah University Hospital, Mount Scopus campus, due to anhydramnios observed during a routine 30-week antepartum checkup. Ultrasound examination revealed a viable fetus with breech presentation and anhydramnios. The fetal bladder was not visualized, while the kidneys appeared to be of normal size and echogenicity. Fetal biometry matched gestational week 29 and fetal weight estimation was 1350 grams (25th percentile by Hadlock). Doppler study of the umbilical and middle cerebral arteries (MCA) was normal (PI 0.57 and 1.6, resp.). The patient denied any unusual vaginal discharge and a speculum examination using Actim Prom kit (Medix Biochemica Espoo, Finland) was negative for preterm premature rupture of the membranes (PPROM). Obstetrical history was remarkable for 7 early miscarriages and one tubal pregnancy. She also had 3 uncomplicated pregnancies that resulted in spontaneous vaginal term deliveries. Her medical history included essential hypertension diagnosed 8 years ago. She was taking Codiovan (valsartan 160 mg + hydrochlorothiazide 12.5 mg Novartis UK) daily, initiated 3 years prior to the current pregnancy. Eye examination, EKG, and renal function at the onset of the pregnancy were all within normal limits. Routine obstetric follow-up, first and second trimester screening for Down's syndrome, and a detailed anatomy scan at 23 weeks' gestation were all normal. The patient did not have any additional ultrasound examinations between gestational weeks 23 and 30, per standard of care.

Upon admission, Codiovan treatment was stopped and the patient was counselled regarding the possible adverse effect of valsartan on fetal renal function. Two weeks later, at 32 weeks' gestation, a follow-up fetal ultrasound showed a normal amniotic fluid index of 7 cm and normal size bladder and kidneys. Fetal biometry was within normal limits as was the Doppler study of the umbilical artery and MCA. Maternal blood pressure was normal, without pharmacological treatment. The patient was then followed up at the outpatient clinic and readmitted at 33 + 4 gestational weeks with PPROM. Four days later, labor was induced due to maternal fever. A viable male weighing 1970 grams was delivered vaginally. Apgar score was 6 and 9 at 1 and 5 minutes, respectively. The newborn was admitted to the neonatal intensive care unit (NICU) for 3 weeks. Blood, urine, and cerebrospinal fluid cultures were all sterile. Newborn renal function was normal from admission until discharge at the age of 3 weeks. Long term follow-up of more than two years has not shown any evidence of renal failure. Serum creatinine at the age of two years was 56 mmol/L.

## 3. Discussion

ACE-I and ARB carry a “black box” warning and have been labeled by the Food and Drug Administration as Pregnancy Category C when used during first trimester of pregnancy and Pregnancy Category D for use during the second and third trimesters. (Drug Information Page, FDA, June 2006). While the teratogenic effects associated with first trimester use remain questionable [7, 12], the adverse effects of these drugs during second and third trimester have been well established. Oligohydramnios due to ARB or ACE-I exposure during second or third trimester has been described in detail [6]. The exact mechanism of fetal renal toxicity in late pregnancy exposure is unknown; however reduced renal perfusion has been suggested as one of the possible causes for permanent renal impairment following exposure to both classes of drugs [13]. Other than oligo- or anhydramnios, detailed sonographic evaluation of affected fetuses usually reveals an enlarged and hyperechogenic kidney. Histological assessment of affected neonates exhibits tubular dysgenesis with absence or poor differentiation of the proximal tubules [14, 15]. These findings usually represent irreversible renal damage and are usually found during postmortem examination.

The typical dosage of valsartan is 80 to 320 mg daily for the treatment of hypertension. The patient we described was taking 160 mg daily, the standard dose for mild, uncomplicated hypertension. One may wish to speculate that a higher dose could have an earlier fetal effect or that the effect would have been irreversible; however, there is no data in the medical literature supporting a dose dependent risk for fetal renal effect.

Oligohydramnios, irrespective of its etiology, if developed during gestational weeks 16 to 22, may lead to fatal pulmonic hypoplasia [16]. This irreversible condition leads to severe respiratory failure and eventually neonatal death, regardless of the gestational age at delivery. The cause of oligohydramnios, whether renal failure, renal agenesis, or early PPROM, does not influence the occurrence or severity of the lung disease. The regain of normal amniotic fluid volume later in pregnancy will usually not reverse the lung pathology. However, if oligohydramnios occurs during the third trimester as often happens due to third trimester PPROM, fetal lungs will not be effected.

There are a handful of case reports and some case series describing adverse pregnancy outcome due to ACE-I and ARB usage during the second and third trimester [17]. This case report is, to the best of our knowledge, one of the few descriptions of reversible fetal renal damage during the third trimester. Since oligohydramnios developed only at about gestational week 30 and resolved several weeks later, the fetus did not develop pulmonary hypoplasia. As hoped, the regain of normal amniotic fluid volume represented recovery of renal function and no signs of renal failure were noted throughout the two-year follow-up.

This case report clearly demonstrates that oligohydramnios noted after gestational week 22, secondary to ACE-I or ARB exposure, can be reversed if the pharmacological agent is stopped. We are reassured that if amniotic fluid levels return to normal, renal function can be preserved, and a healthy infant can develop without sequelae, as evidenced by the two-year follow-up period. This case, like others before, emphasizes the importance of the primary obstetrician awareness regarding the use of teratogenic drugs during pregnancy.
